# Sleep and Haemophilia–A Case‐Control Analysis of Associated Factors

**DOI:** 10.1111/hae.70217

**Published:** 2026-01-30

**Authors:** Alexander Schmidt, Marius Brühl, Pia Möllers, Jamil Hmida, Fabian Tomschi, Joschua Wiese, Heinrich Richter, Jonas Roos, Thomas Hilberg, Andreas Christian Strauss

**Affiliations:** ^1^ Department of Sports Medicine University of Wuppertal Wuppertal Germany; ^2^ Department of Orthopaedics and Trauma Surgery University Hospital Bonn Bonn Germany; ^3^ Haemophilia Centre Muenster Muenster Germany

**Keywords:** haemophilia, haemophilic arthropathy, pain, rare disease, sleep, sleep quality

## Abstract

**Introduction:**

Sufficient sleep is essential for maintaining both physical and mental health, yet data on sleep health among persons with haemophilia (PwH) remain limited.

**Aim:**

This study aimed to assess sleep in PwH compared to healthy controls (Con) and identify factors associated with impaired sleep quality (SQual) in PwH.

**Methods:**

100 PwH A or B and 100 Con participated. Sleep metrics were assessed using the German sleep questionnaire (Schlaffragebogen B revised), comparing SQual, trouble falling asleep (TFA), trouble staying asleep (TSA), feeling of being restored after sleep (RAS), and sleep quantity (SQuan) between groups. Additionally, variables potentially associated with SQual in PwH, i.e. age, BMI, current pain (NRS_now_), pain over four weeks (NRS‐4w), joint health (Haemophilia Joint Health Score (HJHS)), and quality of life (QoL), were analysed using a multiple regression model.

**Results:**

Age‐adjusted comparisons revealed significantly worse SQual (*p* < 0.001) and RAS (*p* = 0.037) in PwH compared to Con, with no significant differences in TFA, TSA, and SQuan. Regression analysis identified only NRS‐4w (*p* = 0.012) and QoL (*p* = 0.008) as significant predictors of worse SQual in PwH (*R* ^2^ = 0.17), while age, BMI, and HJHS had no significant effect.

**Conclusion:**

PwH exhibit poorer SQual and RAS compared to Con, with pain and diminished QoL being key factors contributing to impaired SQual. Other sleep indices such as TFA, TSA and SQuan were unaltered. Given the adverse health impacts of poor SQual and its association with pain, integrating sleep assessment into routine care may enhance patient outcomes.

## 1 Introduction

Haemophilia is a rare congenital coagulopathy characterised by bleeding events that predominantly occur within the musculoskeletal system [1]. These bleedings initiate a cascade of pathophysiological changes, including the upregulation of inflammatory molecules and the degeneration of articular structures [2], resulting in a decline in motor function [3] and diminished psychological well‐being [4]. Besides, a substantial number of persons with haemophilia (PwH) report experiencing chronic pain [5] and a reduction in quality of life (QoL) [6].

Adequate sleep has long been recognised for its system‐preserving effects, maintaining and improving physical and cognitive performance and various health‐related metrics, including blood pressure regulation [7], healthy blood sugar levels [8], and emotional well‐being [9]. Conversely, insufficient sleep has been linked to the development or exacerbation of different metabolic [10], cardiovascular [11] and psychological conditions [12]. Additionally, poor sleep is thought to have a bidirectional relationship with pain, whereby pain can impair sleep quality, and inadequate sleep can amplify pain perception [13]. Current literature indicates that psychological disorders are frequently accompanied by impaired sleep quality [14].

Within the context of haemophilia, research on sleep is limited. To date, only one study has examined the influence of pain on sleep, but it did not assess specific sleep‐related parameters [15]. Consequently, no study to date has specifically explored the sleep profile and its distinct components in PwH. This is a critical gap in the literature, considering the aforementioned health‐related consequences of poor sleep. Therefore, the primary objective of this study was to assess subjective sleep quality and other key sleep‐related outcomes (e.g., trouble falling asleep) in PwH and compare them to healthy individuals. The secondary objective of this study was to examine correlations between factors associated with haemophilia with relevant sleep metrics, as well as to identify determinants of poor sleep in PwH.

## 2 Materials and Methods

### 2.1 Study Design and Participants

This study was carried out as a case‐control, cross‐sectional, questionnaire‐based trial. Data from PwH were collected in person at two centres between April 2022 and October 2024: the University hospital Bonn and the Haemophilia Care Centre in Münster. For the healthy age‐ and gender‐matched control group, questionnaires were digitised and distributed across various social media platforms (e.g., LinkedIn, Instagram). Different application modes between groups were deemed appropriate, given the evidence from a recent systematic review indicated a high inter‐format reliability [16]. To ensure validity of data, control questions were included throughout the different sections of the questionnaire of both the online and paper‐based format for consistency. By means of questionnaires, medical information (e.g., disease severity, treatment regimen), personal data (e.g., date of birth, weight), and information on sleep‐related parameters, pain, and QoL were collected. In addition, the orthopaedic joint status of PwH was assessed utilizing the Haemophilia Joint Health Score version 2.1 (HJHS). All data and informed consent were collected during a single study visit by the same investigator, who had no prior clinical or personal relationship with the participant. Overall, three different investigators were involved in the data acquisition process across the study period. During each visit, PwH first completed the questionnaires. Subsequently, the same investigator conducted the orthopaedic joint assessment. The investigator was partially blinded to the questionnaire results, being blinded to sleep‐ and pain‐related data but not to medical information.

Participants were deemed eligible if they were male, between the ages of 18 and 70 years, and had provided written informed consent. PwH were included irrespective of residual factor activity level (i.e., severe, moderate, or mild) or type of haemophilia (A or B). Exclusion criteria comprised age below 18 or above 70 years, female sex, and the presence of another coagulopathic disease and the occurrence of a bleeding two weeks prior to the examination. Healthy controls were only included if they had no diagnose of any chronic disease (e.g., cardiovascular, metabolic, psychological).

In the online‐based data collection process, participants were informed on the first page about the study's purpose, risks and benefits, confidentiality measures, and the voluntary nature of participation.

### 2.2 Ethics

This study was conducted in accordance with the principles of good clinical and ethical practice and was approved by the local ethics committee (106/21, Bonn). Along the Declaration of Helsinki, participants were informed in detail about the study protocol prior to the examination and were required to give written informed consent. This study was prospectively registered at the Clinical Trials Register (ID: NCT04795921; registration date: 21th of October 2021).

### 2.3 Sleep Questionnaire

Sleep‐related information was gathered using the well‐established and validated German Sleep Questionnaire B revised (SF‐BR) [17]. This self‐report instrument consists of 31 items, mostly rated on a 5‐point Likert scale, designed to assess an individual's sleep behaviour and experiences over the previous two weeks. While the SF‐BR allows for the calculation of five sleep indices and seven factor scales, for the purpose of this study, we selected the sleep indices *trouble falling asleep* (TFA), *trouble staying asleep* (TSA), and *sleep quantity* (SQuan), along with the factor scales *sleep quality* (SQual) and *feeling of being restored after sleep* (RAS). These specific sleep‐related variables were selected as they were considered most relevant within the scope of this investigation, as they provide essential information on sleep disturbances, which may be more pronounced in PwH. For SQual and RAS, higher values indicate better sleep characteristics, whereas for TFA and TSA higher scores reflect worse outcomes. According to cut‐off values provided by the questionnaire manual, SQual, TFA, TSA, and RAS can be classified into different categories based on severity [17].

### 2.4 Quality of Life

Data on QoL were obtained using the generic health‐related Short‐Form 36 questionnaire in German, a validated and widely used instrument in various studies [18]. The questionnaire comprises 36 Likert scale‐based items, clustered into eight distinct dimensions that are further subdivided into a physical and mental component of QoL. For the purpose of this study, both components were pooled to reflect overall QoL. Higher values are indicative of better QoL.

### 2.5 Pain and Joint Status

Participants reported their current intensity of pain (NRS_now_) and their average pain over the past four weeks (NRS_mean_) both on a numerical rating scale ranging from 0 (“no pain”) to 10 (“maximum pain”).

Additionally, the HJHS was used to assess the orthopaedic joint state of the patient group. The ankle, knee, and elbow joints were examined for clinical abnormalities, such as swelling, muscle atrophy, and crepitus. Each individual joint can receive a score of up to 20 points. Considering the possible four score points for gait deviations, the total score can reach a maximum of 124 points, with higher values implying worse joint function [19]. The HJHS was performed by different investigators due to logistical reasons. All examiners were members of the same research group and received identical instructions to ensure the validity of data. Research has also demonstrated excellent interrater reliability [20].

### 2.6 Data Analysis

Statistical analyses and creation of figures were carried out using RStudio (version 4.2.3) with a uniform significance level set at *p* ≤ 0.05. Data distribution was tested analytically using Kolmogorov‐Smirnov tests and graphically by inspection of Q‐Q‐plots. In case of marked violations of normal distribution, parameters were recoded using square root transformations [21]. Data are generally presented as mean and standard deviation (range) or absolute numbers, unless otherwise specified.

In the first step, between‐group comparisons of sleep‐related parameters between PwH and healthy individuals were calculated. Analysis of covariance was employed for the continuous scores and Fisher's exact tests were applied for categorical data. As age was found to differ significantly between the groups, it was included as a covariate in the analyses of continuous variables (e.g., sleep scores). Effect sizes were calculated as partial eta‐squared (*η^2^*_partial_) and interpreted in the following way: *η^2^*_partial_ ≥ 0.01 indicates a small effect, *η^2^*_partial_ ≥ 0.06 indicates a medium effect, *η^2^*_partial_ ≥ 0.14 indicates a large effect [22].

Secondly, Pearson product‐moment correlations were computed to explore the relationship of SQual with age, body‐mass‐index (BMI), NRS_now_, NRS_mean_, HJHS, and QoL in PwH. Pearson correlation coefficients (*r*) were interpreted as follows: *r* < 0.3 indicates a weak correlation, *r* < 0.5 indicates a moderate correlation, and *r* ≥ 0.5 indicates a strong [22].

Lastly, multiple linear regression models were calculated for SQual, incorporating all secondary parameters as predictors to identify the factors most strongly related to poor sleep outcomes in PwH. In cases of heteroscedasticity, the heteroscedasticity‐consistent estimator HC3 was applied to generate robust estimates [23]. Due to concerns regarding the potential presence of multicollinearity between NRS_now_ and NRS_mean_, NRS_now_ was excluded from the regression model for two reasons: first, the correlation coefficient for NRS_now_ was lower, indicating a weaker association with SQual; second, NRS_mean_ captures pain intensity over a prolonged time, which better aligns with the temporally scaled constructs of the SF‐BR. According to the literature, age [24] and BMI [25] are predictive of variations in sleep metrics and were therefore included as covariates in the regression model.

## 3 Results

### 3.1 Participants

In total, data from 100 PwH and 100 healthy controls were obtained and statistically analysed. Personal data for both groups and disease‐specific data for PwH are presented in Table 1.

**TABLE 1:** Characteristics of patients with haemophilia (PwH, *n* = 100) and healthy controls (Con, *n* = 100).

Parameter	PwH	Con	*p*‐value
Age	42.9 ± 14.2 (18.0‐75.0)	37.8 ± 13.1 (18.0‐72.0)	0.010
BMI	25.9 ± 3.7 (19.0‐37.6)	25.3 ± 3.5 (17.1‐36.9)	0.244
Gender (male)	100	100	−
Haemophilia type	Severe A: 73 Severe B: 10 Moderate A: 12 Moderate B: 3 Mild A: 1 Mild B: 1	−	−
Treatment regime	Prophylaxis: 93 On demand: 7	−	−
HJHS	27.1 ± 19.0 (0.0‐85.0)	−	−
NRS_now_	2.1 ± 2.3 (0.0‐8.0)	0.8 ± 1.4 (0.0.‐7.0)	< 0.001
NRS_mean_	2.8 ± 2.1 (0.0‐8.0)	1.4 ± 1.7 (0.0‐7.0)	< 0.001
QoL	47.0 ± 7.3 (29.8‐59.0)	51.3 ± 6.1 (32.9‐58.5)	< 0.001

*Notes*: Data presented as mean ± standard deviation or n. Differences are considered significant for *p* ≤ 0.05, employing Student's t‐tests.

Abbreviations: BMI, body‐mass‐index; HJHS, Haemophilia Joint Health Score version 2.1; NRS_now_, current pain intensity on a numerical rating scale; NRS_mean_, average pain intensity over the past four weeks on a numerical rating scale; QoL, quality of life.

### 3.2 Sleep‐Related Outcomes

**FIGURE 1:**
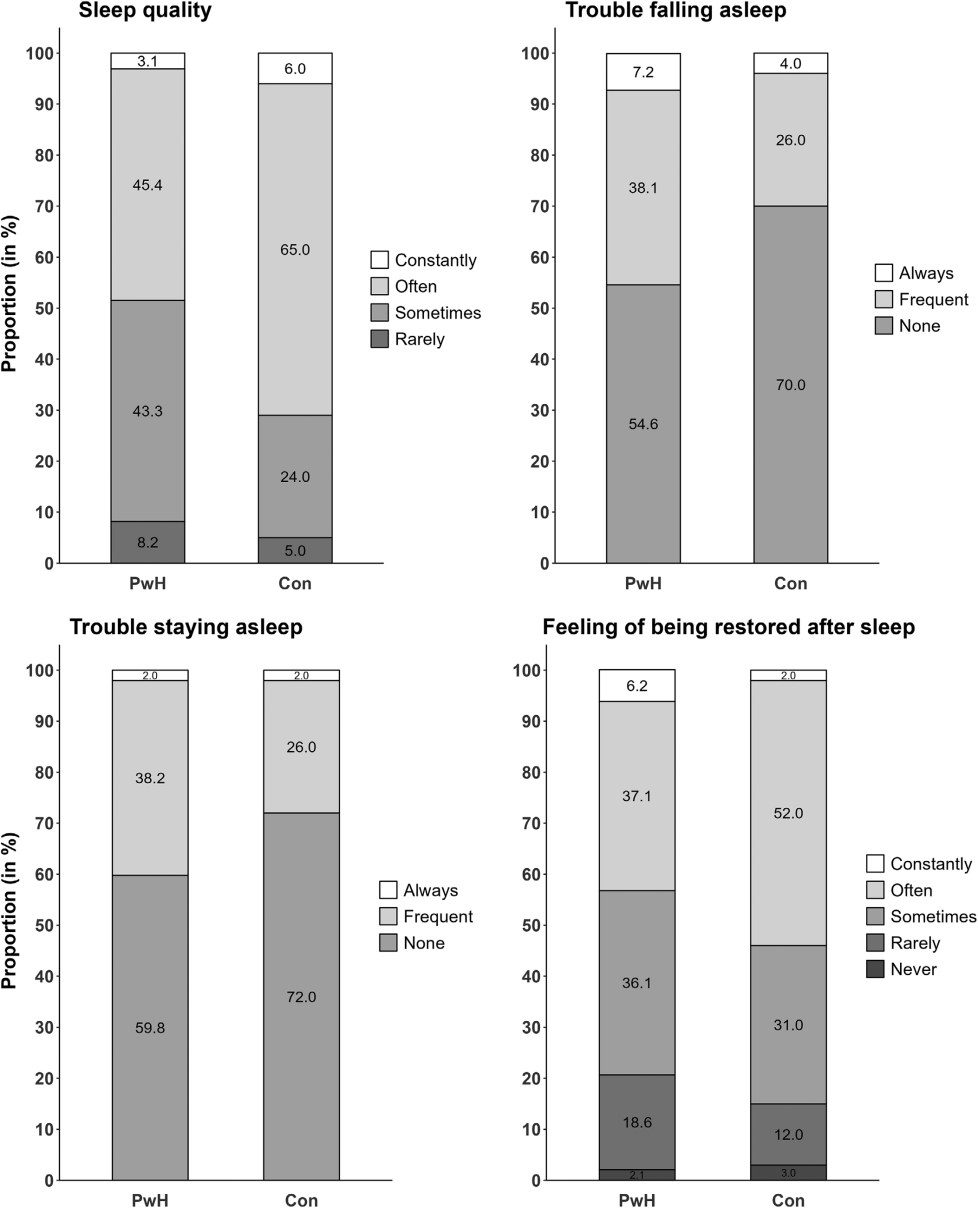
Comparison of sleep characteristics by category between patients with haemophila (PwH, n = 100) and healthy controls (Con, *n* = 100).

Less than 50% of PwH reported consistently good SQual, in contrast to 70.3% among healthy controls. Regarding the sleep parameters TFA and TSA, 45.4% and 40.2% of PwH, respectively, exhibited frequent disturbances, compared to 29.7% and 27.7% among healthy controls. The distribution of RAS showed a similar pattern to that of SQual, with 56.7% of PwH not feeling restored after sleep most of the time (compared to controls: 46.5%). However, based on Fisher's exact tests, significant between‐group differences were observed only for SQual (*p* = 0.014), but not for TFA (*p* = 0.080), TSA (*p* = 0.134), and RAS (*p* = 0.164). Entirety of data on sleep‐related parameters by categories are presented in Figure 1. Additionally, 9% of PwH and 5% of controls reported frequent use of sleep‐promoting agents, with exogenous melatonin being the most commonly used in PwH (*n* = 5/9, 55.6%) and controls (*n* = 2/5, 40%).

**FIGURE 2:**
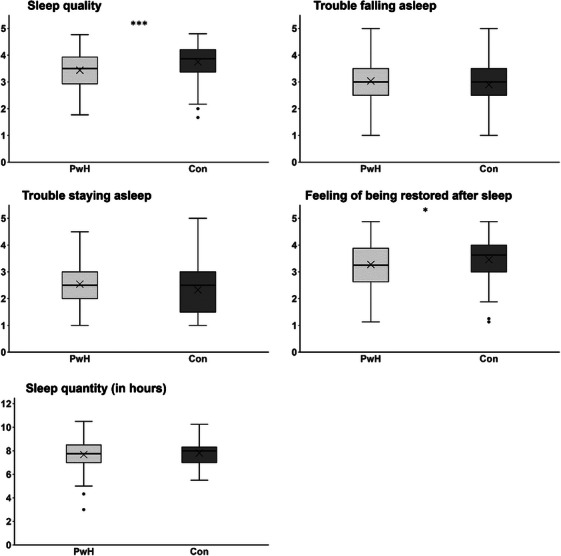
Comparison of sleep metrics in patients with haemophilia (PwH, *n* = 100) and healthy controls (Con, *n* = 100). Boxes indicate data between the 25th and 75th percentiles, with the horizontal bar representing the median and the cross the mean (whiskers: minimum and maximum of data; circles: outliers above/below 1.5 * interquartile range). ***p ≤ 0.001, *p ≤ 0.05, employing analysis of covariance, controlling for age.

Between‐group comparisons, adjusted for age, revealed worse sleep‐related outcomes in PwH in terms of SQual (3.4 ± 0.7) and RAS (3.3 ± 0.8) compared to healthy controls (SQual: 3.8 ± 0.6; *p* < 0.001, *η* ^2^_partial_ = 0.056; RAS: 3.5 ± 0.8; *p* = 0.037, *η* ^2^_partial_ = 0.022). No statistically significant differences between the two study groups were observed for the sleep indices TFA, TSA, and SQuan. Results of the group comparisons can be found in Figure 2, and an overview of the raw data of both groups for all sleep‐related parameters, including effect sizes, is presented in Supplementary Table S1.

### 3.3 Associations With Sleep Quality in PwH

An overview of correlation analyses for PwH is presented in Table 2. In summary, QoL was significantly correlated with SQual (*r* = 0.476, *p* < 0.001), TFA (*r* = ‐0.253, *p* = 0.014), TSA (*r* = ‐0.388, *p* < 0.001), and RAS (*r* = 0.499, *p* < 0.001). NRS_now_ (*r* = ‐0.201, *p* = 0.050) and NRS_mean_ (*r* = ‐0.339, *p* < 0.001) were significantly correlated with SQual. Furthermore, NRS_mean_ (*r* = 0.251, *p* = 0.013) and age (*r* = 0.259, *p* = 0.010) were significantly positively correlated with TSA. For BMI and HJHS no significant correlation with sleep‐related parameters were observed. In healthy controls, SQual (*r* = 0.391, *p* < 0.001), RAS (*r* = 0.498, *p* < 0.001), and TSA (*r* = ‐0.247, *p* = 0.014) showed significant correlations with QoL. Further, age was significantly correlated with RAS (*r* = 0.273, *p* = 0.006) and TSA (*r* = 0.256, *p* = 0.010). No other intercorrelations reached statistical significance in the control group.

**TABLE 2:** Pearson correlation analyses between sleep metrics and age, BMI, pain, clinical joint status and quality of life in patients with haemophilia.

	SQual *r* [95%‐CI] *p*‐value	TFA *r* [95%‐CI] *p*‐value	TSA *r* [95%‐CI] *p*‐value	RAS *r* [95%‐CI] *p*‐value	SQuan *r* [95%‐CI] *p*‐value
Age	*0.055* [−0.146, 0.252] 0.592	−*0.128* [−0.319, 0.073] 0.211	*0.259* [0.063, 0.436] **0.010**	*0.124* [−0.078, 0.315] 0.227	*0.179* [−0.024, 0.367] 0.083
BMI	*0.061* [−0.140, 0.257] 0.554	−*0.071* [−0.267, 0.130] 0.490	*0.060* [−0.141, 0.257] 0.557	−*0.044* [−0.242, 0.156] 0.666	−*0.080* [−0.277, 0.124] 0.442
HJHS	−*0.095* [‐0.289, 0.107] 0.356	*0.062* [−0.140, 0.258] 0.550	*0.110* [−0.092, 0.303] 0.285	−*0.006* [−0.205, 0.193] 0.952	*0.142* [−0.061, 0.334] 0.170
NRS_now_	−*0.201* [−0.386, 0.000] **0.050**	*0.028* [−0.174, 0.227] 0.787	*0.127* [−0.076, 0.319] 0.219	−*0.062* [−0.260, 0.140] 0.547	−*0.042* [−0.242, 0.162] 0.690
NRS_mean_	−*0.339* [−0.505, −0.149] **< 0.001**	*0.151* [−0.051, 0.341] 0.142	*0.251* [0.054, 0.430] **0.013**	−*0.104* [−0.298, 0.099] 0.314	−*0.011* [−0.213, 0.192] 0.918
QoL	*0.476* [0.302, 0.619] **< 0.001**	−*0.253* [−0.434, −0.053] **0.014**	−*0.388* [−0.548, −0.201] **< 0.001**	*0.499* [0.329, 0.637] **< 0.001**	−*0.064* [−0.266, 0.142] 0.543

*Notes*: Differences are considered significant for *p* ≤ 0.05. Significant results are marked in bold.

Abbreviations: BMI, body‐mass‐index; HJHS, Haemophilia Joint Health Score version 2.1; NRS_now_, current pain intensity on a numerical rating scale; NRS_mean_, average pain intensity over the past four weeks on a numerical rating scale; QoL, quality of life; *r*, Pearson correlation coefficient; RAS, feeling of being restored after sleep; SQual, sleep quality; SQuan, sleep quantity; TFA, trouble falling asleep; TSA, trouble staying asleep.

Predictors included within the multiple linear regression model accounted for 17% of the variance in SQual among PwH. NRS_mean_ (*β* = ‐0.088 [95%CI: ‐0.151, ‐0.026], *p* = 0.006) and QoL (*β* = 0.017 [95%CI: 0.005, 0.028], *p* = 0.004) showed significant associations with SQual, whereas age, BMI, and HJHS did not exhibit significant predictive effects. Results of the multiple regression analysis on SQual in PwH are presented in Table 3.

**TABLE 3:** Multiple linear regression analysis in patients with haemophilia. dependant variable: sleep quality (*n* = 100).

					95% CI		
Predictor	B	Robust SE	T	p‐value	LB	UB	VIF
Intercept	2.219	0.478	4.638	< 0.001	1.268	3.169	−
Age	0.006	0.006	1.065	0.290	−0.005	0.018	1.671
BMI	0.019	0.016	1.135	0.260	−0.014	0.051	1.030
HJHS	−0.005	0.005	−1.088	0.280	−0.015	0.004	1.931
NRS_mean_	−0.088	0.032	−2.793	0.006	−0.151	−0.026	1.331
QoL	0.017	0.006	2.933	0.004	0.005	0.028	1.519

*Notes: R^2^* = 0.215; adjusted *R^2^* = 0.170; *F* (8.869) = 4.761, *p* < 0.001.

Abbreviations: BMI, Body‐Mass‐Index; HJHS, Haemophilia Joint Health Score v2.1; NRS_mean_, average pain intensity over the last four weeks on a numerical rating scale from 0 (no pain) to 10 (maximum pain); QoL, quality of life.

## 4 Discussion

This study is the first to address the sleep profile of PwH and aimed to explore factors associated with worse SQual. PwH showed significantly worse SQual and worse RAS than healthy controls, while other sleep outcomes, such as TFA, TSA, and SQuan, were statistically comparable between the groups. Correlation analyses for PwH revealed NRS_now_, NRS_mean_, and QoL to be significantly related to SQual. Besides, QoL was found to be significantly correlated with each other sleep metric, except for SQuan, while NRS_now_ and age were also significantly correlated with TSA. Finally, multiple regression analysis indicated that NRS_mean_ and QoL were the strongest and only significant predictors of poorer SQual in PwH in the model.

Historically, an individual's sleep health was mainly defined by achieving a sufficient SQuan. Our results indicated that PwH and healthy individuals do not significantly differ in total sleep duration. According to our data, 79.0% of PwH meet the recommended 7–9 h of sleep per night for ages 18 to 64 and 7–8 h for those over 64 [26]. These proportions are comparable to those in the general population (74.2%), as reported in a recent systematic review and meta‐analysis involving over a million respondents [27].

In recent years, however, sleep researchers have highlighted the importance of sufficient SQual, often ascribing it greater significance in maintaining overall good health [28, 29], while both adequate SQual and SQuan are proposed to be pivotal for pain regulation [30]. Based on our data, SQual is impaired in PwH compared to healthy controls. This finding is in concordance with research in patients with other musculoskeletal disorders. For instance, a study by Ulus et al. [31] found that patients with rheumatoid arthritis exhibited decreased SQual compared to healthy individuals. Analogously, patients with hip osteoarthritis, particularly those experiencing joint pain, demonstrated worse SQual [32]. According to a recent systematic review on pain prevalence in PwH, 40% of patients reported chronic pain [5], which can be mainly attributed to arthropathic alterations. The high prevalence of pain in PwH has notable sleep‐related consequences, as both NRS_now_ and NRS_mean_ were found to be inversely correlated with SQual. Notably, NRS_mean_ emerged as an overriding determinant of a patients SQual in the presented regression model.

The interrelation between sleep and pain represents a vicious cycle, wherein poor sleep exacerbates pain and vice versa [13]. Furthermore, a study by Abeler and colleagues found the magnitude of self‐perceived pain intensity the next day to be significantly increased in individuals with a poor night sleep [33]. This phenomenon is likely due to the heightened neuronal activity of emotional brain areas associated with pain processing. Additionally, sleep deprivation leads to decreased connectivity to the medial prefrontal cortex, which serves as the control centre for emotional regulation [34]. In addition, current literature suggests that pain thresholds, such as pain sensitivity and pain tolerance, are decreased in both healthy individuals [35] and patient populations with chronic pain [36] who experience sleep impairments. This is noteworthy as pain thresholds represent a more objective aspect of an individual's pain experience. Since the present study focused solely on the subjective nature of pain, future investigations should further elaborate on this association in PwH.

Conversely, and in contrast to the results of this study, literature suggests that pain is associated with insufficient sleep quantity [33] and the higher occurrence of sleep disturbances, typically categorised by prolonged sleep latency and fragmented sleep. When comparing SQuan, TFA, and TSA between patients with and without pain, no significant differences were observed in this study (data not shown). This may be partly attributable to the fact that PwH may adapt to chronic pain over time and cultivate unique coping strategies, as previously proposed [37, 38]. Furthermore, PwH are known to use pain medication to manage chronic pain, evoked mainly by haemophilic arthropathy. The aforementioned coping strategies might also offer a logical explanation for the lack of a significant association between sleep metrics and the observed detrimental joint status (i.e., HJHS). Nevertheless, based on descriptive statistics, PwH reported higher incidences of sleep disturbances than healthy controls. Although the aforementioned sleep parameters did not reveal statistically significant differences, it may be inferred that PwH exhibit a general tendency toward prolonged sleep latency and increased sleep fragmentation.

The results of this study suggest that this susceptibility may be partially explained by increased pain and decreased QoL. As mentioned above, sleep health is closely related to QoL, which was observed to be decreased in PwH in this study and in other research [6]. This notion seems to be applicable to PwH, as QoL was identified as the strongest predictor of poor SQual in this patient group.

From a clinical perspective, these findings highlight the importance of increased awareness of sleep health in the routine care of PwH. Although specific sleep hygiene interventions were beyond the scope of the present study, the observed associations between pain, reduced quality of life, and impaired sleep quality suggest that systematic screening for sleep disturbances may be beneficial in this patient population.

Simple, low‐threshold clinical measures, such as targeted sleep history taking, the use of validated sleep questionnaires, and counselling on basic sleep hygiene principles (e.g. regular sleep–wake schedules, pain management before bedtime, and reduction of sleep‐disrupting behaviours), could represent feasible additions to comprehensive haemophilia care. Early identification of sleep‐related problems may contribute to improved symptom management, enhanced quality of life, and potentially better long‐term outcomes. Future interventional studies are warranted to evaluate whether targeted sleep‐focused strategies can positively influence sleep quality and overall well‐being in PwH.

### 4.1 Strengths and Limitations

The major strength of this study is that it represents the first investigation into sleep health among PwH. In addition to providing descriptive data, sleep‐related parameters were compared to healthy individuals to better contextualise the sleep health of PwH. Furthermore, this study provides valuable insights into factors that negatively impact sleep, which could be targeted to promote improved sleep quality. However, some limitations are worth mentioning. First, sleep metrics were assessed based on self‐report, which may result in overestimation or underestimation of an individuals’ sleep behaviour and experiences. The introduction of objective measures, such as actigraphy or polysomnography, could enhance the validity of data and provide a more in‐depth analysis of sleep outcomes in PwH. However, this investigation collected data merely based on questionnaires as this study served as providing initial insights into the sleep profile of PwH and due to the time‐intensive nature of objective assessments. Additionally, although relevant confounding variables (e.g., age) were acknowledged and controlled for in the statistical analyses, other potentially confounding life‐style factors (e.g., diet, physical activity) as well as pain medication or comorbidities were not included. Future research should consider these variables to provide a more holistic understanding of sleep dynamics in PwH. Lastly, although age was statistically controlled for in the analyses, a residual confounding effect related to the significant age differences between groups cannot be entirely excluded. However, sleep‐related parameters, such as SQual, do not deteriorate upon reaching specific age thresholds; rather, they follow a progressive decline across the entire lifespan and are individually determined [39].

## 5 Conclusion

In comparison to healthy individuals, PwH demonstrated impaired sleep quality and a lower sense of restfulness following sleep, while other sleep parameters – including difficulties initiating of maintaining sleep and total sleep quantity – remained comparable between groups. Sleep quality in PwH was particularly affected by pain and reduced quality of life. Given the negative impact of reduced sleep quality in various health metrics and its bidirectional relationship with pain, healthcare professionals should routinely assess sleep quality during clinical consultations. Furthermore, patient education on sleep‐promoting behaviours should be integrated into standard care to support overall health and pain management.

## Author Contributions

**Alexander Schmidt**: data curation, investigation, writing – original draft, methodology, writing – review and editing, visualization, formal analysis, software. **Marius Brühl**: investigation, writing – review and editing. **Pia Möllers**: investigation, writing – review and editing, conceptualisation, data curation. **Jamil Hmida**: investigation, writing – review and editing, data curation, conceptualisation. **Fabian Tomschi**: writing – review and editing, formal analysis, software. **Joschua Wiese**: investigation, writing – review and editing. **Heinrich Richter**: writing – review and editing, resources. **Jonas Roos**: investigation, writing – review and editing. **Thomas Hilberg**: project administration, conceptualisation, supervision, writing – review and editing, resources. **Andreas Christian Strauss**: project administration, conceptualisation, supervision, writing – review and editing, resources.

## Funding

The authors have nothing to report.

## Ethics Statement

The study protocol was approved by the local Ethics Committee (Friedrich–Wilhelms–Universität Bonn, 106/21) and was conducted in accordance with the Declaration of Helsinki. All participants provided written informed consent.

## Consent

Participants signed the informed consent according to the Helsinki Declaration Statement.

## Conflicts of Interest

AS has received speaker's fees from Swedish Orphan Biovitrum and Takeda. MB has received travel costs from Swedish Orphan Biovitrum and Takeda. PM has received speaker's fees and travel support from Swedish Orphan Biovitrum and Takeda. JH has no competing interests to declare. FT has received speaker's fees from Takeda and received an educational grant from Swedish Orphan Biovitrum. JW has received speaker's and travel fees from Takeda. HR has received research and travel grants from Bayer, Biotest, CSL Behring, Intersero, Novo Nordisk, Pfizer, Swedish Orphan Biovitrum and Takeda. JR has no competing interests to declare. TH has received research funding from Biotest, Chugai, CSL Behring, Intersero, Roche, Swedish Orphan Biovitrum and Takeda as well as travel expenses, speaker's or scientific advisory board honoraria from Bayer, Biotest, Chugai, Novo Nordisk, Pfizer, Roche, Sanofi, Sobi and Takeda. ACS has received research funding from Bayer, Swedish Orphan Biovitrum and Takeda and has received consultancy, speaker's bureau, honoraria, scientific advisory board and travel expenses from Bayer, Biotest, CSL Behring, Novo Nordisk, Swedish Orphan Biovitrum and Takeda.

## Supporting information




**Supporting Table 1**: Sleep metrics in patients with haemophilia (PwH, *n* = 100) and healthy controls (Con, *n* = 100).

## Data Availability

The data that support the findings of this study are available on request from the corresponding author upon reasonable request.
