# Supplementary material for: Sleep and Haemophilia–A Case‐Control Analysis of Associated Factors
Source: Haemophilia. 2026 Jan 30;32(2):471–80. doi: 10.1111/hae.70217 (PMC12984463; doi:10.1111/hae.70217)
Supplement: Supplementary file 1 — Supporting Table 1: Sleep metrics in patients with haemophilia (PwH, n = 100) and healthy controls (Con, n = 100). [file HAE-32-471-s001.docx]

**Supplementary Table 1.** Sleep metrics in patients with haemophilia (PwH, n=100) and healthy controls (Con, n=100)

| **Sleep metric** | **PwH** | **Con** | **p-value** | **Effect size** |
| --- | --- | --- | --- | --- |
| SQual | 3.44 ± 0.67 | 3.75 ± 0.61 | < 0.001 | 0.056 |
| TFA | 3.04 ± 0.87 | 2.90 ± 0.75 | 0.185 | 0.009 |
| TSA | 2.54 ± 0.84 | 2.33 ± 0.84 | 0.251 | 0.007 |
| RAS | 3.28 ± 0.80 | 3.46 ± 0.79 | 0.037 | 0.022 |
| SQuan | 7.67 ± 1.17 | 7.80 ± 0.90 | 0.360 | 0.004 |

Data presented as mean ± standard deviation. Differences are considered for p ≤ 0.05, employing Student’s t-tests. RAS = feeling of being restored after sleep, SQual = sleep quality, SQuan = sleep quantity, TFA = trouble falling asleep, TSA = trouble staying asleep.
